# Phase I dose escalation study of telatinib (BAY 57-9352) in patients with advanced solid tumours

**DOI:** 10.1038/sj.bjc.6604724

**Published:** 2008-11-11

**Authors:** D Strumberg, B Schultheis, I A Adamietz, O Christensen, M Buechert, J Kraetzschmar, P Rajagopalan, M Ludwig, A Frost, S Steinbild, M E Scheulen, K Mross

**Affiliations:** 1 Department of Haematology and Medical Oncology, University of Bochum (Marien Hospital, Herne), Herne, Germany; 2 Department of Radiotherapy, University of Bochum (Marien Hospital, Herne), Herne, Germany; 3 Bayer Pharmaceuticals Inc., Montville, NJ, USA; 4 Magnetic Resonance Development and Application Centre, University Hospital, Freiburg, Germany; 5 Bayer Schering Pharma, Wuppertal, Germany; 6 Tumour Biology Centre at the Albert-Ludwigs-University, Freiburg, Germany; 7 Department of Internal Medicine and Medical Oncology, West German Cancer Centre, University of Essen, Germany

**Keywords:** phase I, telatinib, vascular endothelial growth factor receptor, tyrosine kinase inhibitor, pharmacodynamics, pharmacokinetics

## Abstract

Telatinib (BAY 57-9352) is an orally available, small-molecule inhibitor of vascular endothelial growth factor receptors 2 and 3 (VEGFR-2/-3) and platelet-derived growth factor receptor *β* tyrosine kinases. In this multicentre phase I dose escalation study, 71 patients with refractory solid tumours were enroled into 14 days on/7 days off (noncontinuous dosing) or continuous dosing groups to receive telatinib two times daily (BID). Hypertension (23%) and diarrhoea (7%) were the most frequent study drug-related adverse events of CTC grade 3. The maximum-tolerated dose was not reached up to a dose of 1500 mg BID continuous dosing. Telatinib was rapidly absorbed with median *t*_max_ of 3 hours or less. Geometric mean *C*_max_ and AUC_0−12_ increased in a less than dose-proportional manner and plateaued in the 900–1500 mg BID dose range. Two renal cell carcinoma patients reached a partial response. Tumour blood flow measured by contrast-enhanced magnetic resonance imaging and sVEGFR-2 plasma levels decreased with increasing AUC_0−12_ of telatinib. Telatinib is safe and well tolerated up to a dose of 1500 mg BID continuous dosing. Based on pharmacokinetic and pharmacodynamic criteria, 900 mg telatinib BID continuously administered was selected as the recommended phase II dose.

Telatinib (BAY 57-9352) is an orally available, potent, small-molecule inhibitor of vascular endothelial growth factor (VEGF) receptors 2 and 3 (VEGFR-2/-3) and platelet-derived growth factor (PDGF) receptor *β* (PDGFR-*β*) tyrosine kinases.

The growth of solid tumours is accompanied by angiogenesis (Algire *et al*, 1945) and the development of an effective vascular network is required for the tumours to grow beyond 1 *μ*l in volume (Folkman, 1990). Anti-angiogenesis is an interesting strategy for the treatment of cancer (Folkman, 1971). VEGF and its receptor, VEGFR-2, which is expressed on activated endothelial cells associated with growing solid tumours, are required for the angiogenic process (Folkman, 2002; Pisacane and Risio, 2005). Continued signal transduction through the VEGF/VEGFR-2 pathway is a primary stimulus for initiation and maintenance of tumour angiogenesis (Ellis, 2004). Blocking the interaction of VEGF with the VEGFR-2 receptor or inhibiting the tyrosine kinase activity of the VEGFR-2 receptor blocks both angiogenesis and tumour growth in *in vivo* models. Complete suppression of tumour growth has been demonstrated using dominant-negative VEGF receptors (Zhang *et al*, 1995; Millauer *et al*, 1996; Goldman *et al*, 1998) and blocking antibodies (Kim *et al*, 1993; Asano *et al*, 1995; Warren *et al*, 1995; Melnyk *et al*, 1996; Yuan *et al*, 1996; Borgstrom *et al*, 1998) as well as small-molecule inhibitors of VEGFR-2 kinase (Wood *et al*, 2000; Wedge *et al*, 2002) as single-agent therapies in model systems. Overexpression of VEGF is common in solid tumours and associated with poorer prognosis (Dosquet *et al*, 1997; Raben and Helfrich, 2004; Giatromanolaki *et al*, 2006).

Telatinib is a potent inhibitor of VEGFR-2 and PDGFR-*β* tyrosine kinase activity measured in a biochemical assay. These two receptors play key roles in the angiogenic process involving the stimulation of endothelial cells and PDGFR-expressing pericytes. Telatinib inhibited VEGFR-2 autophosphorylation in a whole-cell assay of receptor autophosphorylation *in vitro*, VEGF-dependent proliferation of human umbilical vein endothelial cells *in vitro*, and PDGF-stimulated growth of human aortic smooth muscle cells. Telatinib demonstrated potent, dose-dependent reduction in tumour growth *in vivo* in a variety of models including MDA-MB-231 breast carcinoma, Colo-205 colon carcinoma, DLD-1 colon carcinoma and H460 non-small cell lung carcinoma. Toxicological studies supported the start of a clinical study in cancer patients at a dose level of 10 mg (once daily (OD)) of telatinib.

The N-methyl group of telatinib was identified as the main target of metabolic degradation. The *in vitro* investigations using human microsomes, hepatocytes or single cytochrome P450 (CYP) isoforms revealed that there is no or only a very low risk of drug–drug interactions. Telatinib was metabolised by various CYP isoforms. There was no critical involvement of polymorphic CYP isoforms in the biotransformation. Telatinib exhibited neither an inhibitory nor an inductive potential on major human CYP isoforms at therapeutically relevant concentrations. Drug–drug interactions are also unlikely to occur due to displacement from plasma protein-binding sites or modulation of p-glycoprotein transporter activity based on the results of *in vitro* studies.

This phase I clinical study had the objective to determine the dose-limiting toxicities (DLTs), maximum-tolerated dose (MTD) and pharmacokinetics of oral telatinib. Preliminary antitumour activity, interaction with a variety of biomarkers including VEGFR-2 and dynamic contrast-enhanced magnetic resonance imaging (DCE-MRI) were evaluated.

## Patients and methods

### Patient selection

Eligible patients were ⩾18 years of age, with a life expectancy of at least 12 weeks, and a solid tumour that was refractory to standard treatment or without standard therapy options. Patients had to have Eastern Cooperative Oncology Group (ECOG) performance status of 0–1. All patients had evaluable disease according to the Response Evaluation Criteria in Solid Tumours (RECIST) criteria. Patients might have had any number of prior systemic therapy, radiotherapy or surgery, but therapies had to be discontinued at least 4 weeks before study entry (6 weeks in case of mitomycin C and nitrosoureas).

Other eligibility criteria included the following: (1) adequate haematopoietic (absolute neutrophil count (ANC) ⩾1.5 × 10^9^ l^−1^; platelet count ⩾150 × 10^9^ l^−1^ and haemoglobin ⩾9.0 g dl^−1^), hepatic (total bilirubin ⩽1.5 times the upper limit of normal (ULN); aspartate aminotransferase and alanine aminotransferase ⩽2.5 times ULN; prothrombin time and international normalised ratio of partial thromboplastin time <1.5 times ULN unless on therapeutic anticoagulants), and renal (serum creatinine ⩽1.5 times ULN) functions; (2) no pregnancy and breast feeding; (3) no clinically relevant co-morbidity such as cardiovascular diseases and no clinically relevant co-medication; (4) no metastatic brain or meningeal tumours, unless the patient was >6 months from definitive therapy and had a negative imaging study within 4 weeks of study entry.

All patients provided written informed consent in accordance with federal and institutional guidelines before study treatment.

### Study design

This was a multicentre, open-label, non-controlled, phase I dose escalation study to investigate the safety, pharmacokinetics and pharmacodynamics of oral telatinib (BAY 57-9352). Administration of telatinib was continued until an unacceptable toxicity, disease progression or death occurred or the consent was withdrawn. At start of the study, only a solution formulation was available. The formulation as tablet was introduced into the study after first pharmacokinetic results became available. Based on pharmacokinetic data, OD, two times daily (BID), and three times daily schedules were evaluated. For the sake of clarity, the data presented in this paper refer to the patients enroled into the BID 14 days on/7 days off (noncontinuous dosing) and continuous dosing groups only.

Three patients were initially enroled at each dose level. If no DLT had occurred at the end of the 3-week treatment cycle, three patients were enroled at the next dose level. If any patient experienced a DLT, three additional patients were enroled at that dose level. If at least two out of six patients experienced a DLT, dose escalation had to be stopped and that dose was to be declared the toxic dose. The next lower dose level was defined as the MTD. DLTs were defined as grade 3 or 4 non-haematological toxicities, febrile neutropenia (ANC <0.5 × 10^9^ l^−1^ and fever ⩾38.5°C), grade 4 neutropenia lasting for at least 7 days, platelet count <25 × 10^9^ l^−1^ or grade 3 or 4 thrombocytopaenic bleeding, which occurred during cycle 1. In the course of the study the protocol was amended so that the onset of CTC grade 3 hypertension was only considered to be a DLT if the hypertension turned out to be refractory to standard antihypertensive treatment. The number of patients enroled per dose level was extended to six patients for the dose levels of 150 mg BID or higher to get more reliable estimates for telatinib pharmacokinetic parameters.

Adverse events were assessed at the end of each cycle and graded according to the National Cancer Institute Common Toxicity Criteria (NCI CTC), v2.0 (Trotti *et al*, 2000).

### Patient evaluation

History, physical examinations, haematological and biochemical laboratory evaluations were performed at screening, on days 1, 7 and 14 of cycle 1 and on day 1 of subsequent cycles. Baseline objective tumour measurements were performed within 4 weeks prior to study treatment. Lesions at all disease sites were categorised as either measurable or nonmeasurable. Indicator lesions were selected and monitored throughout the study by the same assessor and using the same technique. Tumour response was evaluated according to the RECIST (Therasse *et al*, 2000).

### Pharmacokinetics

Patients with at least one valid pharmacokinetic profile were valid for the pharmacokinetic analysis. Plasma samples were collected at predose and 0.5, 1, 2, 3, 4, 6, 8, and 12 h postdose on day 1 and day 14 of cycle 1 and were analysed for BAY 57-9352 and its demethylated metabolite M-2, BAY 60-8246, using a validated LC-MS-MS analytical method.

Plasma pharmacokinetic parameters, area under the curve from time 0–12 h after dosing (AUC_0−12_), area under the curve from time 0 to last data point (AUC_0−tn_), maximum plasma concentration (*C*_max_), and time to maximum plasma concentration (*t*_max_) of telatinib and its metabolite (BAY 60-8246) as well as half-life of telatinib were calculated by non-compartmental methods using WinNonlin version 4.1.a (Pharsight Corporation). The linear-logarithmic trapezoidal rule was used for calculating AUC. Half-life was calculated by linear least squares regression after logarithmic transformation of the terminal concentrations. Pharmacokinetic parameters were analysed using descriptive statistics.

### Pharmacodynamics

The effects of telatinib treatment on the plasma concentrations of sVEGFR-2, VEGF and bFGF were determined from blood samples taken at baseline, on day 14 of cycles 1, 2, 4, 6, etc. and at the final visit. Samples were analysed using the relevant quantitative enzyme-linked immunosorbent assay (ELISA) (R&D Systems Europe, Oxford, UK) according to the manufacturer's instructions. DCE-MRI was performed at baseline, on day 2 (only cycle 1), and on day 14 of cycles 1–3 to assess tumour blood flow/tumour vessel permeability in a subgroup of patients (Morgan *et al*, 2003).

## Results

### Patient characteristics

A total of 71 patients (30 women, 41 men) with refractory advanced solid tumours were enroled into the BID noncontinuous and continuous treatment groups. Patients' median (range) age was 60 (31–82) years, median (range) weight 73.6 (39–113) kg. Baseline ECOG performance status was 0 in 36 patients (51%), 1 in 32 patients (45%) and 2 in 2 patients (3%). For one patient, no baseline ECOG performance status was documented. The main tumour types were colorectal cancer (*n*=25, 35%), renal cell carcinoma (*n*=12, 17%), hepatocellular carcinoma (*n*=9, 13%), non-small cell lung cancer and pancreatic cancer (both *n*=4, 6%). 56 patients (79%) had prior systemic anticancer therapy, 63 (93%) prior anticancer surgery, and 20 (28%) prior radiotherapy. All 71 patients were valid for safety and pharmacokinetic analyses.

### Dose escalation and MTD

Dose escalation started with a single oral dose of 10 mg telatinib. The starting dose was based on nonclinical data. Based on the pharmacokinetic results of the first three patients, which showed a considerably lower than expected exposure, multiple dosing was initiated at 20 mg OD. Doses of 20–300 mg telatinib OD were administered for 14 days followed by 7 days off treatment. At doses of 150 and 300 mg OD, no further increase in exposure to telatinib was achieved. As safety and tolerability remained good, BID dosing was initiated at 75 mg BID using the same noncontinuous regimen of 14 days followed by 7 days off treatment. For the sake of clarity, the data presented in this paper refer to the patients enroled in the BID-dosing cohorts only. Up to 1500 mg BID in the noncontinuous regimen, only two patients experienced DLTs, that is, grade 3 hypertension, at doses of 300 and 1500 mg BID, respectively. A further dose escalation beyond the 1500 mg BID dose level was not feasible due to the number of tablets to be taken. As the MTD had not been reached for the noncontinuous treatment, the continuous BID dosing was initiated at 600 mg BID. Dose escalation was stopped at 1500 mg BID continuous dosing without reaching the MTD of telatinib.

The results of the BID noncontinuous and continuous dosing groups are reported here. Telatinib was administered as solution and 25 mg mesylate tablet in the 75 mg BID noncontinuous dosing group, as solution, 25 and 150 mg mesylate tablets, and 150 mg base tablet in the 150 mg BID noncontinuous dosing group, as 25 mg mesylate tablet in the 300 mg BID noncontinuous dosing group, and as 150 mg tablet in all other groups. Table 1 shows an overview about the dose escalation steps and the treatment duration. Fifteen patients were enroled at the 150 mg BID dose level as the relative bioavailability for different tablet formulations was evaluated at this dose level.

### Safety

In total, 21% of all patients experienced at least one adverse event assessed by the investigators as study drug-related with worst CTC grade of 1–2 and 25% at least one study drug-related adverse event with worst CTC grade 3 (Table 2). There were no study drug-related adverse events of CTC grades 4 or 5 reported in this study.

The most common toxicity was hypertension in 4% of the patients with worst CTC grade 1–2 and in another 23% of the patients with worst CTC grade 3 (Table 2). Grade 3 hypertension occurred in one-third to half of the patients in the 600–1500 mg noncontinuous dosing groups and the 900 mg continuous dosing group (Table 2). In the 1200 mg continuous dosing group, more than two-thirds of the patients experienced grade 3 hypertension. In most cases hypertension was clinically well manageable with a standard antihypertensive treatment. In three patients at dose levels of 300 mg BID, 1500 mg BID noncontinuous dosing and 1200 mg BID continuous dosing, hypertension resulted in dose reduction and dose interruption, in one of them finally to permanent discontinuation of study drug treatment.

Other common adverse events were gastrointestinal toxicities such as anorexia and diarrhoea (Table 2). Diarrhoea led to dose reduction or interruption in four patients at dose levels of 900 mg BID or higher, in one of them to permanent discontinuation. One patient at the 1500 mg BID continuous dosing level had a dose interruption due to nausea and vomiting.

Serious study drug-related adverse events (adverse events leading to hospitalisation or assessed by the investigator as medically important) occurred in five patients: two patients had diarrhoea (dose levels: 1500 mg BID noncontinuous and 1200 mg BID continuous dosing), two patients had hypertension (dose levels: 1500 mg BID noncontinuous and 1200 mg BID continuous dosing), and one patient experienced a hand–foot skin reaction and dehydration (dose level: 900 mg BID continuous dosing).

Dose-limiting toxicities were reported for two patients (dose levels: 300 mg BID and 1500 mg BID noncontinuous dosing). Both had hypertension refractory to standard treatment leading to dose reduction of telatinib. As at the highest dose level administered in this study, 1500 mg BID continuous dosing, no patient out of six patients experienced dose-limiting toxicities within the first 21 days of treatment, the MTD was not reached in this study.

### Pharmacokinetics

**Figure 1:**
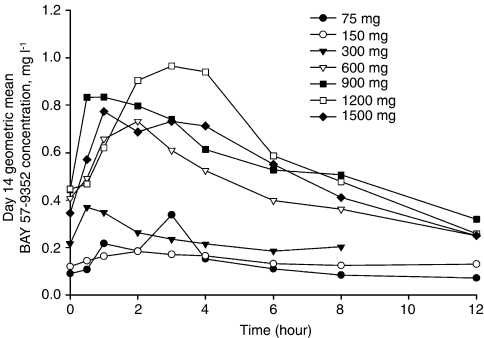
Geometric mean telatinib plasma concentration *vs* time profiles on day 14 of cycle 1.

Day 14 steady-state geometric mean (percent coefficient of variation) telatinib and BAY 60-8246 pharmacokinetic parameters are shown in Table 3 and day 14 geometric mean telatinib plasma concentration *vs* time profiles are shown in Figure 1. For the 150 mg BID dose level, pharmacokinetic results were available from different exploratory formulations. For this dose level, results from only the 25 mg telatinib mesylate tablet formulation are shown in Table 3 and Figure 1.

Following oral administration, telatinib was rapidly absorbed with median *t*_max_ of 3 h or less in the 75 mg BID to 1500 mg BID dose range. Geometric mean *C*_max_ increased in a less than dose-proportional manner in the dose range of 75 mg BID to 300 mg BID. Geometric mean *C*_max_ increased two-fold between the 300 and 600 mg BID dose level and subsequently increased in a less than dose-proportional manner up to 1500 mg BID. Although a reason for the two-fold increase in geometric mean *C*_max_ is not known, it is not attributable to the 150 mg telatinib mesylate tablet formulation. Bioavailability assessments performed with the 25 and 150 mg tablets indicated that the relative bioavailability of the 150 mg tablet formulation is less when compared with the 25 mg tablet formulation. Increases in telatinib geometric mean AUC_0−tn_ and AUC_0−12_ followed a pattern similar to that described for *C*_max_. Less than dose-proportional increase was observed in the 600–1500 mg BID dose range. In general, exposure was comparable in the 900–1500 mg BID dose range. These results (and relevant biomarker results) formed the basis for choosing 900 mg BID as the recommended phase II dose for telatinib. The geometric mean half-life at the 900 mg BID dose range was 5.6 h thus supporting the BID-dosing regimen.

After oral administration of telatinib, maximum concentrations of the metabolite (BAY 60-8246) were observed approximately around the same time as the parent compound or shortly thereafter. Plasma concentrations of BAY 60-8246 were generally lower when compared with telatinib plasma concentrations. At the recommended phase II dose of 900 mg BID, geometric mean *C*_max_ and AUC_0−12_ values of the metabolite were less than 20% of the corresponding geometric mean *C*_max_ and AUC_0−12_ values of the parent compound. Less than dose-proportional increase observed with the parent compound was also observed with the metabolite. These results and the results of the mass balance study conducted in healthy subjects (data not shown) indicate that BAY 60-8246 is of minor importance in humans.

### Pharmacodynamics

To assess the biological activity of telatinib, plasma concentration analyses for the angiogenic markers VEGF, sVEGFR-2, bFGF, PDGF and IL-6 were performed at baseline and during the course of the study. Furthermore, DCE-MRI measurements were done at baseline, on days 2 and 14 of cycle 1 and on day 14 of cycles 2 and 3. Evaluable DCE-MRI results were available for a subgroup of patients treated at dose levels of 300 mg BID or higher.

VEGF plasma levels showed a dose-dependent short-term increase within 8 h after the first telatinib administration. VEGF levels increased also comparing day 21 to baseline. sVEGFR-2 levels showed a dose-dependent decrease over the course of the study. In addition, a decrease in the iAUC60 for the gadolinium curve as measured by DCE-MRI was observed.

**Figure 2:**
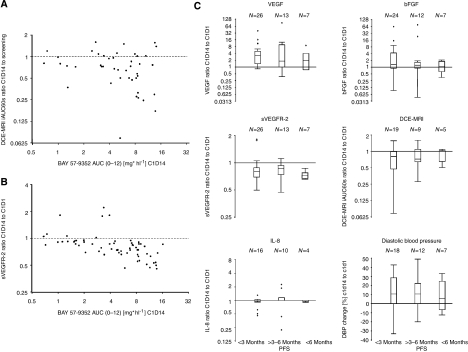
Analysis of telatinib AUC_0−12_ on day 14 of cycle 1 *vs* the ratio of the initial 60 s area under the gadolinium curve (iAUC60) on day 14 of cycle 1 to the iAUC60 at baseline (**A**) and *vs* the ratio of sVEGFR-2 in plasma on day 14 of cycle 1 to sVEGFR-2 at baseline (**B**); correlation of pharmacodynamic parameters to progression-free survival (**C**).

The analysis of telatinib AUC_0−12_ on day 14 of cycle 1 *vs* the ratio of gadolinium iAUC60 on day 14 of cycle 1 to iAUC60 at baseline is shown in Figure 2A. In general, the gadolinium iAUC60 ratio decreased with increasing telatinib AUC_0−12_ although a statistically significant correlation between telatinib exposure and the pharmacodynamic effect as measured by DCE-MRI was not observed (regression *r*^2^=0.0625; Pearson correlation coefficient *ρ*=−0.250; test for no correlation, H_0_: *ρ*=0, *P*=0.10). Substantial decreases in the gadolinium iAUC60 ratio were observed at total daily doses of ⩾600 mg telatinib corresponding to telatinib AUC_0−12_ values of about 4 mg h l^−1^.

The analysis of telatinib AUC_0−12_ on day 14 of cycle 1 *vs* the ratio of sVEGFR-2 in plasma on day 14 of cycle 1 to sVEGFR-2 at baseline is shown in Figure 2B. The ratio of sVEGFR-2 in plasma decreased with increasing telatinib AUC_0−12_, that is, essentially in an exposure-dependent manner (regression *r*^2^=0.2973; Pearson correlation coefficient *ρ*=−0.545; test for no correlation, H_0_: *ρ*=0, *P*=0.0001).

To correlate biomarker changes to the clinical outcome, the patients were categorised into those who had a progression-free survival of <3 months, 3 up to 6 months, or >6 months. The relative changes between cycle 1, day 14 and baseline were calculated for VEGF, sVEGFR-2, bFGF, IL-8, tumour blood flow and tumour vessel permeability as measured by DCE-MRI and diastolic blood pressure (Figure 2C). Changes from baseline were observed for plasma VEGF and sVEGFR-2 levels, the decrease in tumour blood flow and permeability and also for the increase in diastolic blood pressure. The bFGF and IL-8 plasma levels showed no relevant changes after 14 days of multiple dosing with telatinib. The changes in VEGF and sVEGFR-2 plasma levels, the decrease in tumour blood flow and permeability and the increase in diastolic blood pressure were not predictive for the clinical outcome; there were no statistically significant differences in the change of biomarkers for patients who reached a progression-free survival of >3 months compared with those who stopped treatment during the first 3 months due to progressive disease.

### Efficacy

Table 4 summarises the best tumour responses according to RECIST, study duration and medication days on telatinib by tumour type. Seventy-one patients were assessable for tumour response. Patients with renal cell carcinoma (RCC, *n*=12) showed the most promising preliminary antitumour activity: two of them reached a partial response, and the median treatment duration for patients with RCC was 164 days compared with 89 days in the overall study population.

## Discussion

Telatinib is safe and well tolerated up to doses of 1500 mg BID continuous dosing. The most frequent study drug-related adverse events were hypertension and gastrointestinal toxicities such as anorexia and diarrhoea. The treatment with telatinib had to be dose reduced or discontinued permanently in only nine out of 71 patients due to drug-related averse events. At the highest dose level administered in this study, 1500 mg BID continuous dosing, none of the six patients experienced a DLT within the first 21 days of treatment, whereas at 1500 mg BID noncontinuous dosing, one out of six patients experienced a DLT, that is, grade 3 hypertension refractory to standard treatment. The MTD was not reached in this study.

The safety profile of telatinib is comparable to other small-molecule VEGFR-inhibiting compounds. Hypertension as a common class toxicity phenomenon was clinically well manageable in most of the patients with a standard antihypertensive treatment. Recently, Steeghs *et al* (2008) reported that small vessel rarefaction may be one of the underlying haemodynamic mechanisms causing hypertension. The average increase in diastolic blood pressure in our study was comparable to those reported results.

In our study, diarrhoea led to dose reductions in three patients. The occurrence of gastrointestinal toxicities is also known for other VEGF-inhibiting compounds (Escudier *et al*, 2007; Motzer *et al*, 2007). The variability of pharmacokinetic parameters was significant. Geometric mean exposure to telatinib increased in a less than dose-proportional manner up to 1500 mg BID. In general, exposure was similar in the 900–1500 mg BID dose range. Thus further increase in dose did not result in a further increase in drug exposure. The short half-life of 6.6–10.9 h was the reason for BID administration of telatinib.

The biomarkers assessed in this study demonstrated the biological activity of telatinib. The angiogenic factors VEGF and sVEGFR-2 showed effects known from other VEGF-inhibiting compounds. Increases in VEGF and decreases in sVEGFR-2 were dose-dependent and correlated to telatinib exposure. The DCE-MRI parameters Ktrans and iAUC60 showed a proof of mechanism for telatinib. However, there was no correlation between the clinical outcome and the biomarker activity. This might be due to the heterogeneous study population and the various dose levels used in this study.

The safety profile of telatinib was acceptable and a toxic dose level with two out of six or more DLTs at one dose level was not reached in this study even at the highest dose of 1500 mg BID continuously administered. A further dose escalation was not feasible due to the number of tablets to be taken at these high dose levels and the pharmacokinetic data showed that an exposure plateau was reached at dose levels of 900 mg BID or higher. In concordance with the pharmacokinetic exposure, the pharmacodynamic data revealed no additional effects beyond the 900 mg BID dose level. Taking the tolerability, pharmacokinetic and biomarker data into consideration, the recommended phase II dose level for single-agent telatinib is 900 mg BID administered continuously.

The treatment with telatinib showed anticancer effects in two patients with RCC who reached a partial remission. RCC is one of the tumour types most sensitive to VEGF-inhibiting therapeutics (Escudier *et al*, 2007; Motzer *et al*, 2007).

In conclusion, telatinib is an orally available small-molecule inhibitor of VEGFR-2/-3 and PDGFR-*β* tyrosine kinases with a favourable safety profile in patients with refractory advanced solid tumours. The observed antitumour activity and pharmacodynamic results warrant further evaluation of telatinib in patients with advanced cancer. The recommended phase II dose of telatinib is 900 mg BID as continuous dosing based on pharmacokinetic data, the toxicity profile and the biomarker evaluations.

## Figures and Tables

**Table 1:** Treatment summary by treatment group and dose level (all patients, *N*=71)

**Treatment group**	***N***	**Duration (medication days) (median (range)**
*Noncontinuous dosing (14 days on/7 days off)*		
75 mg BID	4	106 (35–182)
150 mg BID	15^a^	77 (5–633)
300 mg BID	8	58.5 (14–287)
600 mg BID	6	77.5 (35–525)
900 mg BID	6	156.5 (55–497)
1500 mg BID	6	58.5 (35–387)
		
*Continuous dosing*		
600 mg BID	7	35 (11–216)
900 mg BID	6	120.5 (64–164)
1200 mg BID	7	160 (14–178)
1500 mg BID	6	83 (35–120)

BID=two times daily.

a At the 150 mg BID dose level, the relative bioavailability of different tablet formulations was assessed. Therefore the data from 15 patients were pooled for this analysis.

**Table 2:** Incidence of patients (*N*⩾2 (⩾3%)) with study drug-related adverse events with worst CTC grades 1–2 and incidence of patients with study drug-related adverse events with worst CTC grade 3^a^ (*N*(%); all patients, *N*=71)

**NCI CTC category**	**Noncontinuous dosing (mg BID)**				**Continuous dosing (mg BID)**				**Total**
**NCI CTC term CTC grades 1–2**	**75–300 (*N*=27)**	**600 (*N*=6)**	**900 (*N*=6)**	**1500 (*N*=6)**	**600 (*N*=7)**	**900 (*N*=6)**	**1200 (*N*=7)**	**1500 (*N*=6)**	***N*=71**
*Any category*									
Any event	2 (7)	2 (33)	3 (50)	2 (33)	1 (14)	2 (33)	1 (14)	2 (33)	15 (21)
									
*Cardiovascular*									
Hypertension			1 (17)	1 (17)				1 (17)	3 (4)
									
*Constitutional symptoms*									
Fatigue		2 (33)							2 (3)
									
*Gastrointestinal*									
Anorexia	1 (4)	1 (17)	1 (17)	2 (33)					5 (7)
Diarrhoea, patients without colostomy				1 (17)	1 (14)			2 (33)	4 (6)
Flatulence							1 (14)	1 (17)	2 (3)
Nausea				1 (17)		1 (17)		1 (17)	3 (4)
Vomiting				1 (17)				1 (17)	2 (3)
									
*Pulmonary*									
Voice changes/stridor/larynx		2 (33)	2 (33)			1 (17)			5 (7)
									
*Dermatology/skin*									
Alopecia	1 (4)					1 (17)			2 (3)
									
**CTC grade 3** a									
*Any category*									
Any event	1 (4)	3 (50)	2 (33)	4 (67)		3 (50)	5 (71)		18 (25)
									
*Cardiovascular*									
Hypertension	1 (4)	3 (50)	2 (33)	3 (50)		2 (33)	5 (71)		16 (23)
									
*Gastrointestinal*									
Dehydration						1 (17)			1 (1)
Diarrhoea, patients without colostomy				1 (17)		1 (17)	2 (29)		4 (6)
Diarrhoea, patients with colostomy						1 (17)			1 (1)
									
*Pain*									
Abdominal pain or cramping				1 (17)			1 (14)		2 (3)
									
*Dermatology/skin*									
Hand–foot skin reaction						1 (17)			1 (1)
Rash/desquamation						1 (17)			1 (1)

BID=two times daily; NCI CTC=National Cancer Institute Common Toxicity Criteria.

a There were no study drug-related adverse events of CTC grades 4 or 5 reported in this study.

**Table 3:** Geometric mean (%CV) day 14 telatinib and BAY 60–8246 pharmacokinetic parameters after oral administration of telatinib

*Telatinib*							
Dose	75 mg BID	150 mg BID	300 mg BID	600 mg BID	900 mg BID	1200 mg BID	1500 mg BID
*n*	4	6	6	11	12	6	12
Mesylate tablet	25 mg	25 mg	25 mg	150 mg	150 mg	150 mg	150 mg
*C*_max_ (mg l^−1^)	0.212 (140%)	0.219 (80%)	0.389 (153%)	0.810 (87%)	1.275 (57%)	1.144 (40%)	0.964 (83%)
*t*_max_ (h)^a^	2.1 (2.0–3.1)	3.0 (0.52–5.5)	1.5 (0–8.5)	2.1 (1.0–4.2)	2.0 (0.5–8.1)	2.5 (1–4.3)	2.5 (0.6–6.0)
AUC_0–tn_ (mg h l^−1^)	1.38 (150%)	1.75 (84%)	1.92 (142%)	5.43 (68%)	7.41 (51%)	7.10 (31%)	6.17 (80%)
AUC_0–12_ (mg h l^−1^)	1.39 (146%)	1.73 (81%)	2.86 (176%)^b^	5.43 (68%)	7.30 (52%)	7.26 (31%)	6.29 (84%)
*t*_1/2_ (h)	7.4 (33%)	10.9 (66%)^†^	8.0 (58%)^b^	8.7 (46%)	5.6 (62%)^b^	5.6 (100%)	6.6 (65%)
							
*BAY 60–8246*							
*C*_max_ (mg l^−1^)	0.02 (202%)	0.018 (89%)	0.021 (133%)	0.084 (140%)	0.216 (74%)	0.226 (87%)	0.102 (147%)
*t*_max_ (h)^a^	3.0 (0.5–6.0)	2.5 (1.0–12.3)	1.5 (0–8.5)	2.1 (0.6–10)	3.7 (1.0–8.6)	4.0 (2.1–4.3)	3.5 (0.5–6.1)
AUC_0–tn_ (mg h l^−1^)	0.125 (333%)^c^	0.157 (74%)	0.115 (113%)^b^	0.650 (128%)	1.27 (79%)	1.66 (81%)	0.74 (155%)
AUC_0–12_ (mg h l^−1^)	0.153 (232%)	0.155 (72%)	0.219 (58%)^b^	0.650 (128%)	1.25 (80%)	1.71 (84%)	0.76 (160%)

AUC_0–12_=area under the plasma concentration versus time curve from time 0–12 h; AUC_0–tn_=area under the plasma concentration versus time curve from time 0 to last data point; BID=two times daily; *C*_max_=maximum plasma concentration; %CV=percent coefficient of variation; *t*_max_=time to reach maximum plasma concentration; *t*_1/2_=terminal half-life.

a Median (range).

b Sample size reduced by 2.

c Sample size reduced by 1.

**Table 4:** Best response by tumour type and overall response according to RECIST

**Primary tumour site**	***N***	**Study duration (days)**		**Medication (days)**		**Best response**					**Stabilisation (months)**		
		**Median**	**Range**	**Median**	**Range**	**PR**	**SD**	**PD clin**	**PD meas**	**NA**	**<6**	**>6**	**>12**
Colorectal	25	96	21–618	79	16–497		17	1	6		23	1	1
RCC	12	164	17–612	161	14–525	2	7	1	1		10		2
HCC	9	81	15–634	65	14–633		5	4			8	1	
NSCLC	4	116	57–197	106	56–160		3		1		4		
Pancreatic	4	50	15–107	43	14–105			2	2		4		
Other	17	83	6–389	77	5–387		10	1	4	1	13	3	
Total	71	89	6–634	79	5–633	2	42	9	14	1	62	5	3

HCC=hepatocellular carcinoma; NA=not assessable for response; NSCLC=non-small-cell lung cancer; PD clin=progressive disease, clinical judgement; PD meas=progressive disease, measurement proven; PR=partial response; RCC=renal cell carcinoma; RECIST=Response Evaluation Criteria in Solid Tumours; SD=stable disease.
